# Breastfeeding Practices and Problems Among Obese Women Compared with Nonobese Women in a Brazilian Hospital

**DOI:** 10.1089/whr.2021.0021

**Published:** 2021-06-29

**Authors:** Marina Rico Perez, Lucíola Sant'Anna de Castro, Yan-Shing Chang, Adriana Sañudo, Karla Oliveira Marcacine, Lisa H Amir, Michael G. Ross, Kelly Pereira Coca

**Affiliations:** ^1^ Escola Paulista de Enfermagem, Universidade Federal de São Paulo, São Paulo, Brazil.; ^2^ Department of Preventive Medicine, Escola Paulista de Medicina, Universidade Federal de São Paulo, São Paulo, Brazil.; ^3^ Florence Nightingale Faculty of Nursing, Midwifery and Palliative Care, King's College London, London, United Kingdom.; ^4^ Judith Lumley Centre, La Trobe University, Victoria, Australia.; ^5^ Breastfeeding Service, Royal Women's Hospital, Victoria, Australia.; ^6^ Obstetrics and Gynecology, Geffen School of Medicine at UCLA, Los Angeles, California, USA.

**Keywords:** breastfeeding, maternal obesity, body mass index, early weaning

## Abstract

***Background:*** Women who are obese have lower rates of breastfeeding initiation and duration and are less likely to breastfeed exclusively compared with women who are not obese. To develop programs to improve breastfeeding practices among this group of women, we investigated the association between maternal obesity and breastfeeding practices and problems in the first days postpartum.

***Methods:*** We analyzed medical records from postpartum women at a rooming-in maternity ward in State of São Paulo, Brazil, between 2016 and 2018. We included those who had intended to exclusively breastfeed, had given birth to a singleton and were admitted to rooming-in. We analyzed exclusive breastfeeding and nonexclusive breastfeeding each day of hospitalization and the presence of breastfeeding problems, comparing women in the obese category (body mass index [BMI] ≥30 kg/m^2^) to normal and overweight women (≥18.6 to ≤29.9 kg/m^2^).

***Results:*** Two hundred and twenty-four postpartum women participated, including 86 women in the obese category. More than 50% of women with obesity reported a breastfeeding problem in the first and second postpartum days (*p* = 0.026 and *p* = 0.017, respectively) compared with the 41% and 38% nonobese group. Children of obese women were 2.8 times more likely to have poor latch during breastfeeding (95% confidence interval [CI]: 1.29–6.10) compared with the nonobese group on the third day.

***Conclusion:*** Maternal obesity increased the probability of breastfeeding difficulties and nonexclusive breastfeeding at discharge. Professionals need to support breastfeeding techniques in the days immediate after delivery to improve breastfeeding outcomes for mothers with obesity.

## Introduction

The World Health Organization (WHO) reported that obesity was one of the major public health problems globally, as the worldwide prevalence of obesity nearly tripled between 1975 and 2016.^1^ In Brazil, obesity rates have increased among all age groups, in both sexes and at all income levels, although with a greater growth rate in the population with lower schooling.^2^ Among women of reproductive age, obesity rates increased from 12% in 2006 to 21% in 2019 according to the latest Brazilian Health data.^2^

Despite the benefits and exclusive breastfeeding recommendations of health organizations, only 37% of infants were exclusively breastfed during the first 6 months worldwide,^3^ including Brazil (38.6%).^4^ Breastfeeding rates are lower in women who are obese compared with women who are not obese. Women with prepregnancy body mass index (BMI) ≥30 kg/m^2^ are less likely to intend to exclusively breastfeed compared with normal weight and overweight women (78.8% vs. 95.5% and 96.2%, respectively).^5^ The lower rate of breastfeeding intention is critical because maternal intention to breastfeed is among the strongest factors associated with length of lactation.^6^

Compared with women who are not obese, women who are obese have lower rates of initiation and shorter duration of breastfeeding throughout the first year after delivery, and are less likely to breastfeed exclusively, even with adjustment of confounding variables (*e.g.*, maternal age, parity, type of delivery, smoking, intention to breastfeed,^7,8^ and previous history of delayed lactogenesis).^9,10^ An Australian cohort study reported that women who are obese have nearly one-half the probability to initiate breastfeeding and they are 1.4 times less likely to continue breastfeeding at 6 months.^11^ Despite the reported lower rates of breastfeeding in obese mothers,^12^ encouragement of exclusive breastfeeding (EBF) may improve the rates of EBF at 6 months.^13^ Obese women also have higher chances of earlier formula supplementation, are at increased risk for early weaning,^5,6,11,14^ and early breastfeeding cessation.^15^

Several reasons have been proposed to explain the relationship between obesity and the lower rates of breastfeeding, including mechanical issues (breast engorgement, larger amounts of adipose tissue, flattened areolas, and edema) and hormonal factors such as delayed lactogenesis.^16–18^ Obese women are more likely to report problems with milk supply because of their changes in prolactin levels,^19^ hypoplasia of the mammary gland and reduced stromal tissue.^20^ Other risk factors associated with obesity include delayed early contact as a consequence of cesarean section^18,21^ and poor body image.^22^ Women who are obese experience both physical and psychological barriers to the initiation and continuation of breastfeeding.^6,23^

Considering the global trend of increasing obesity in both general and obstetric populations, the benefits of breastfeeding for women and children, and the challenges experienced by puerperal women who are obese, it is of particular importance to provide adequate care to this population to facilitate the initiation maintenance, and exclusivity of breastfeeding.^24^ Because breastfeeding practices are strongly influenced by maternal feeding intention, we aimed to study women who intended to exclusively breastfeed to investigate the specific problems they encounter in the immediate postpartum period.

## Methods

### Study design and setting

**FIG. 1.**
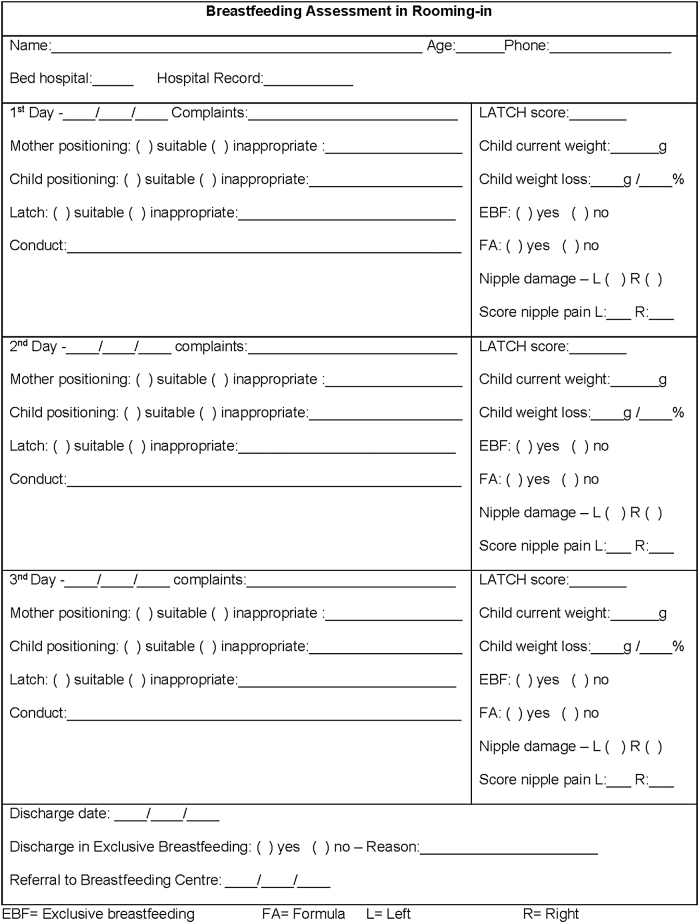
Mother–baby breastfeeding assessment form.

A retrospective cohort study was conducted with the medical records of women who gave birth between June 2016 and September 2018 in the public maternity unit of a hospital located in the city of São Paulo, Brazil (Sao Paulo hospital). The maternity unit is a referral site for high-risk pregnant women and has an average of 52 births per month, with a total of 1401 births during the study period. The routine of hospital lactation consultants was to assess mother–baby breastfeeding performance at least once a day from birth to hospital discharge (Fig. 1). On the second day after delivery, the nutritionist weighed and measured the height of the women on a calibrated digital scale with a coupled stadiometer. All data were recorded on the mother's and infant's medical records.

### Participants

**FIG. 2.**
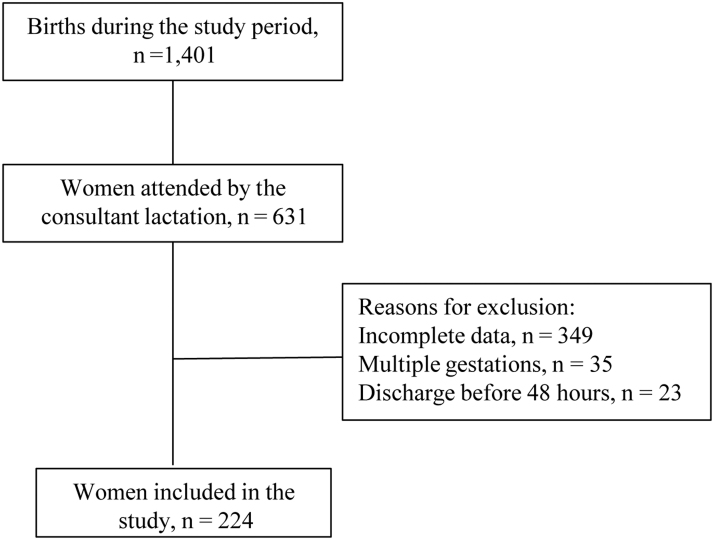
Flowchart of sample selection.

Eligible study criteria included complete medical records from the postpartum women who were attended by the lactation consultant at least once a day during their postpartum hospital stay and had intended to exclusively breastfeed on admission to the postpartum ward. We included births ≥34 weeks' gestation, infants ≥2000 g, who were clinically stable and were rooming-in. We excluded women who were underweight (BMI <18.5 kg/m^2^), had inverted nipple/s, multiple gestations, or hospital stays <48 hours (Fig. 2).

### Data collection

Data were extracted from maternal and infant medical records by lactation consultants. Maternal sociodemographic characteristics (age, schooling, and had a partner), health conditions (postpartum body weight, height, presence of diabetes, and/or hypertension before or after pregnancy), and obstetric data (parity and type of delivery) were obtained from maternal medical records. From infant's medical records, newborn data (gestational age, birth weight, skin-to-skin contact at birth, and early breastfeeding [breastfeed within the first hour after birth]) were obtained. Maternal medical records also were extracted for clinical examination on breastfeeding (assessment of nipple damage, nipple pain, mother and child positioning, baby's latch, and prescription of formula supplementation by pediatrician at the time of discharge).

### Data measures

Participants were classified according to BMI: underweight (BMI <18.5 kg/m^2^), normal (BMI ≥18.6 and ≤24.9 kg/m^2^), overweight (BMI ≥25 and ≤29.9 kg/m^2^), or obese (BMI ≥30 kg/m^2^)^25^ based on measures at day 2 postpartum. We compared women with obesity to the combined group of overweight and normal weight women. We considered skin-to-skin contact at birth when it was reported in the medical records that the newborn was placed naked in direct contact with the mother's breast skin, as soon as s/he was born or shortly thereafter.^26^ Early breastfeeding was defined as the baby having her/his first suckling within the first hour after birth.

Feeding practices were classified according to the WHO (2009)^27^: EBF (when the infant received only breast milk), and non-EBF (when the infant received infant formula in at least one feeding) in the previous 24 hours. The mother and child positioning, and baby's latch were analyzed using the Portuguese version^28^ of the original LATCH Scoring System^29^ that provides a systematic assessment for latch, audible swallowing, type of nipple, comfort, and hold. The system assigns a numerical score (0, 1, or 2) to these five key components of breastfeeding (maximum 10 points). A higher score indicates better feeding attributes.

We defined breastfeeding problems when the lactation consultant indicated that a mother had one of the following: visual evidence of nipple damage observed by the lactation consultant, nipple pain reported by the women during a breastfeeding observation session, poor mother and child positioning during a breastfeed, or poor latch as assessed by the lactation consultant using the LATCH tool.^28^

### Statistical methods/analysis

Descriptive data are presented as measures of central tendency (mean) and dispersion (standard deviation [SD] and minimum and maximum values) for quantitative variables, and relative and absolute frequencies for qualitative values. Chi square, Fisher's exact test, and Student's *t*-tests were used to compare the groups.

The variables of outcome (feeding practices, problems related by the mother, nipple damage and pain, mother and child positioning/latch) were analyzed by Generalized Linear Models with panel data using the “xtlogit “(Stata/SE 15.1 for Windows—StataCorp). All models included group effect (obese or nonobese), time (days 1, 2, and 3) and the interaction between group and time. The results are expressed as odds ratio and respective 95% confidence interval (95% CI). In all analysis, a significance level of *p* = 0.05 was adopted.

The Research Ethics Committee of the university approved the project under No. 1.814.160/2016, according to the guidelines and norms of the National Health Council Resolution (No. 466/2012).

## Results

Two hundred and twenty-four postpartum women were included in the study (Fig. 2). The average age was 31 years (SD = 6.4), average education of 11 years (SD = 2.3), and 91% of women reported a partner. In this sample, 38.4% of women were obese. Among all women, ∼37% had hypertension or diabetes mellitus in pregnancy. Two thirds (67.6%) of women were multiparous and slightly more than half (52.3%) had a vaginal birth. The average gestational age was 38.2 ± 1.4 weeks (range 34–41 weeks) and average birth weight was 3170 g (SD = 482). Overall, 60.7% of babies had skin-to-skin contact at birth and 41.6% had early breastfeeding.

Table 1 presents women and baby's characteristics according to maternal BMI (obese or nonobese). There were no statistically significant differences between the groups in age, schooling, reported partner, proportion primiparous, and vaginal delivery. Obese mothers had a higher frequency of diabetes mellitus and/or hypertension when compared with nonobese women (*p* < 0.001). There were no statistically significant differences in infant weight, term delivery, skin-to-skin contact at birth, or rates of early breastfeeding.

**Table 1.** Sociodemographic, Obstetric, and Neonatal Characteristics by Body Mass Index Groups

Variables	Groups				*p*
	Obese^b^ (*N* = 86)		Nonobese^b^ (*N* = 138)		
	*n*	%	*n*	%	
Women					
Age (year)^a^	31.8 (5.9)		30.8 (5.9)		0.283
Schooling (years of study)^a^	11.1 (2.6)		11.1 (2.2)		0.968
Has a partner	77	89.5	126	92.0	0.535
DM/SAH^c^	47	54.6	35	25.4	<0.001
Primiparous	28	32.6	44	32.4	0.975
Vaginal delivery	42	50.6	73	53.3	0.699
Pregnancies^a^	2.9 (1.7)		2.5 (1.5)		0.088
Newborn					
Birth weight (grams)^a^	3222.2 (485.8)		3138.9 (479.6)		0.209
≥37 weeks gestational age	73	85.9	124	91.2	0.218
Skin-to-skin contact at birth	47	62.7	75	59.5	0.659
Early breastfeeding	34	42.0	55	41.4	0.929

São Paulo, 2016/2018.

^a^ Average (standard deviation).

^b^ Body mass index.

^c^ Diabetes mellitus and/or Systemic Arterial Hypertension in pregnancy.

Table 2 presents measures for the breastfeeding outcomes and breastfeeding problems evaluated daily during hospital stay. By the third postpartum day, 83% of nonobese women reported EBF compared with 64% of obese women (*p* = 0.053). Obese women had >50% greater chance of reporting a breastfeeding problem in the first and second postpartum days (*p* = 0.026 and *p* = 0.017, respectively) compared with nonobese group. Infants of obese women were 2.8 times more likely to have a poor latch during breastfeeding (95% CI: 1.29–6.10) on the third day. There were no differences for the other variables analyzed.

**Table 2.** Body Mass Index Groups, Breastfeeding Practices, and Breastfeeding Problems During Hospital Stay

	Group									
	Obese		Nonobese		Comparison obese × nonobese			Effect of obesity at days 2 and 3		
	*n*	%	*n*	%	OR	95% CI	*p*	OR	95% CI	*p*
EBF										
Day 1	56/79	70.9	107/132	81.1	0.54	0.28–1.04	0.065	—	—	—
Day 2	56/83	67.5	103/134	76.9	0.62	0.34–1.13	0.119	1.13	0.56–2.28	0.721
Day 3	27/42	64.3	60/72	83.3	0.43	0.18–1.01	0.053	0.79	0.32–1.98	0.621
BF problems										
Day 1	44/77	57.1	52/126	41.3	1.91	1.08–3.39	0.026	—	—	—
Day 2	45/82	54.9	51/134	38.1	1.97	1.13–3.43	0.017	1.03	0.50–2.10	0.941
Day 3	19/42	45.2	29/72	40.3	1.13	0.53–2.40	0.754	0.59	0.24–1.43	0.241
Nipple damage										
Day 1	12/78	15.4	25/128	19.5	0.77	0.37–1.63	0.496	—	—	—
Day 2	23/82	28.1	50/135	37.0	0.67	0.37–1.21	0.185	0.87	0.41–1.85	0.715
Day 3	21/40	52.5	33/72	45.8	1.21	0.59–2.52	0.598	1.58	0.65–3.84	0.316
Nipple pain										
Day 1	10/78	12.8	23/128	18.0	0.67	0.30–1.48	0.318	—	—	—
Day 2	15/81	18.5	37/134	27.6	0.60	0.31–1.17	0.135	0.90	0.39–2.07	0.798
Day 3	15/40	37.5	22/71	31.0	1.46	0.68–3.13	0.327	2.19	0.85–5.66	0.106
Inadequate position of mother										
Day 1	44/78	56.4	63/126	50.0	1.27	0.72–2.24	0.407	—	—	—
Day 2	29/82	35.4	58/133	43.6	0.71	0.40–1.25	0.230	0.56	0.27–1.14	0.112
Day 3	8/41	19.5	24/72	33.3	0.53	0.22–1.29	0.162	0.42	0.16–1.13	0.085
Inadequate position of baby										
Day 1	54/76	71.1	73/123	59.4	1.65	0.90–3.03	0.108	—	—	—
Day 2	41/82	50.0	55/133	41.4	1.44	0.83–2.50	0.200	0.87	0.41–1.84	0.716
Day 3	20/42	47.6	25/72	34.7	1.67	0.78–3.57	0.190	1.01	0.40–2.53	0.982
Poor latch										
Day 1	47/78	60.3	68/128	53.1	1.28	0.73–2.27	0.389	—	—	—
Day 2	38/82	46.3	62/133	46.6	0.99	0.57–1.72	0.973	0.77	0.37–1.59	0.482
Day 3	26/42	61.9	26/72	36.1	2.81	1.29–6.10	0.009	2.19	0.88–5.43	0.092

São Paulo, 2016/2018.

EBF, exclusive breastfeeding; BF, breastfeeding; CI, confidence interval; OR, odds ratio.

The mean LATCH score was higher (*i.e.*, better) in nonobese women compared with women with obesity (7 vs. 6; *p* = 0.004) on the first postpartum day, but the difference did not persist on following days. At the time of hospital discharge, obese women (27%) had a greater probability of non-EBF than nonobese women (13.5%; *p* = 0.017).

## Discussion

In this study, the majority of obese women intending to exclusively breastfeed reported breastfeeding problems during hospitalization and were not exclusively breastfeeding at hospital discharge. Newborns of obese mothers demonstrated a greater chance of poor latch compared with those of nonobese mothers during the hospital stay.

As noted previously, the initiation of breastfeeding in obese women^5^ may be adversely impacted by a delay in lactogenesis,^10,18,30^ discomfort with body image and lower self-confidence,^10,18,31,32^ and large breast size.^16,33^ Breastfeeding cessation is often attributed to breast problems, including nipple pain/damage, and mother–baby poor position and latch.^34–37^ Consistent with these reports, our study demonstrated that obese women had greater breastfeeding problems compared with nonobese women in the immediate postpartum period. Specifically, obese women were more likely to experience interruption of breastfeeding due to nipple pain/damage and consequences of poor latch. The larger breast size may cause a difficultly for mothers to visualize and her newborn to correctly latch.^16,33,38^ In our study, obese women had lower LATCH score than nonobese women, confirming prior reports.^39^ Obese women were more likely to report non-EBF at discharge compared with nonobese women, in agreement with the findings of de Jersey et al.^40^ A retrospective study of obese postpartum women demonstrated that EBF at hospital discharge was less prevalent among obese women,^5,41,42^ and that early breastfeeding problems adversely impacted the rate of EBF.

Our study indicates the necessity of providing tailored breastfeeding support for obese mothers.^43–45^ Among the entire maternity population, breastfeeding education and support is associated with higher rates of breastfeeding initiation.^46^ Support for obese women in first days after birth^23^ may enable them to continue breastfeeding after hospitalization.^16,47,48^

Health professionals, partners, and family members need to be prepared to support obese women.^23^ As there is limited evidence regarding the effectiveness of educational and physical breastfeeding support for this group of women, further study is needed to develop and evaluate effectiveness of obese-specific breastfeeding support programs,^49^ including social support (emotional and psychological)^50–52^ and physical support (provision of breast pumps).^52^ Others have also suggested the value of demonstration of multiple feeding positionings such as laid-back or side-lying,^53^ support of breastfeeding with large breasts,^33^ and continued support after hospital discharge^54^ in providing breastfeeding support to obese women.

The limitations of this study include the comparatively small sample size from a single health service site and the lack of longer-term follow-up data.

## Conclusion

Maternal obesity increased the probability of breastfeeding difficulties and non-EBF at hospital discharge. Health care professionals are encouraged to support breastfeeding techniques in the first days after birth to enable obese women to exclusively breastfeed as intended.

Considering the significant increase worldwide in the proportion of the population with high BMIs, this study highlights the importance of promoting enhanced lactation care in the immediate postpartum period to obese women. There is an urgent need to design, develop, validate, implement, and disseminate efficient strategies to support breastfeeding in this population to improve the health of the mother–child dyad and avoid the use of breast milk substitutes in the immediate newborn period.

## Abbreviations Used

BF: breastfeeding
BMI: body mass index
CI: confidence interval
EBF: exclusive breastfeeding
OR: odds ratio
SD: standard deviation
WHO: World Health Organization
